# Percutaneous coronary intervention in diabetic versus non-diabetic patients with prior coronary artery bypass grafting: a propensity score matching study

**DOI:** 10.1186/s12872-020-01447-8

**Published:** 2020-04-06

**Authors:** Xiaolong Ma, Ran Dong, Pengfei Chen, Yichen Zhao, Caiwu Zeng, Meng Xin, Qing Ye, Jiangang Wang

**Affiliations:** 1 Department of Cardiac Surgery, Beijing Anzhen Hospital, Capital Medical University, Beijing, China; 2 Center for Cardiac Intensive Care, Beijing Anzhen Hospital, Capital Medical University, Beijing, China

**Keywords:** Percutaneous coronary intervention, Diabetic, Non-diabetic, Prior coronary artery bypass grafting, Outcomes

## Abstract

**Background:**

The target of this study was to explore the outcomes of percutaneous coronary intervention (PCI) in diabetic versus non-diabetic patients with prior coronary artery bypass grafting (CABG) surgery.

**Methods:**

Seven hundred and twenty four patients who had previously received CABG and had been treated using PCI combined with drug-eluting stents (DES) between 2009 and 2017 were selected for a retrospective study and allocated into either a diabetes mellitus (DM) or non-diabetes mellitus (No DM) group. A 1:1 propensity score-matched evaluation was conducted and risk adjusted for analysis. The primary outcomes were cardiac death, myocardial infarction, heart failure and revascularization, with a median follow-up duration of 5.13 years.

**Results:**

After matching, two-, 5- and 8-year event rate of overall major adverse cardiac events (MACEs) were found to be higher in the DM group (No DM vs DM:15.3, 30.9, 38.5% vs 19.8, 37.8, 52.2%, respectively), although no significant difference was found in the event rate of overall MACEs (hazard ratio [HR]: 1.35; 95% confidence interval [CI]: 1.00 to 1.83 for DM vs No DM; *P* = 0.052), cardiac death (HR: 0.94; 95% CI: 0.45 to 1.95; *P* = 0.871), MI (HR: 1.49; 95% CI: 0.95 to 2.32; *P* = 0.080), HF (HR: 1.54; 95% CI: 0.90 to 2.63 for; *P* = 0.120) or revascularization (HR: 1.07; 95% CI: 0.72 to 1.59; *P* = 0.747). Subgroup analysis of PCI in only the NCA showed MACEs (adjusted HR: 1.13; 95% CI: 0.85 to 1.49 for DM vs No DM; *P* = 0.325), cardiac death (adjusted HR: 0.85; 95% CI: 0.41 to 1.78 for DM vs No DM; *P* = 0.781), MI (adjusted HR: 1.32; 95% CI: 0.84 to 2.01 for DM vs No DM; *P* = 0.069), HF (adjusted HR: 1.41; 95% CI: 0.87 to 2.27 for DM vs No DM; *P* = 0.211) or repeated revascularization (adjusted HR: 0.93; 95% CI: 0.64 to 1.37 for DM vs No DM; *P* = 0.836).

**Conclusions:**

Compared with non-diabetic patients with prior CABG, subsequent implantation of DES in the native coronary artery of diabetic patients resulted in apparently similar outcomes.

**Trial registration:**

This study was not registered in an open access database.

## Background

Diabetic patients have a higher prevalence of coronary artery disease (CAD) than the general population, manifesting as diffuse lesions and severe atherosclerosis in the left main artery and multiple other vessels [1], severe symptoms often developing earlier in life combined with a substantially poorer prognosis than non-diabetic patients [2, 3]. It has been reported that diabetes is considered a predictor of adverse events such as myocardial infarction (MI), repeat revascularization and cardiac death for patients who have undergone coronary artery bypass grafting (CABG) [4–6].

For patients with prior CABG who require repeat revascularization, percutaneous coronary intervention (PCI) is usually the preferred strategy, rather than redo CABG, because of the low procedural mortality and similar long-term outcome [7, 8], combined with placement o f a drug-eluting stent (DES) [9]. Despite a number of studies investigating the impact that diabetes has on the clinical outcome of PCI with DES in patients without prior CABG [10, 11], little is known about the influence of diabetes on outcomes of PCI with DES in patients who have previously undergone CABG.

In this study, we retrospectively assessed the clinical data of non-diabetic and diabetic patients with prior CABG who had subsequently received PCI with DES, aiming to establish the impact of DM on the long-term outcomes of PCI for restenosis after CABG.

## Methods

### Study design

This study was a retrospective observational study conducted in Beijing Anzhen Hospital, Capital Medical University, Beijing Institute of Heart Lung and Blood Vessel Diseases, Beijing, China. A total of 724 patients with prior CABG were selected from the institution’s PCI registry (2009 to 2017) who had undergone PCI with DES in a native coronary artery (NCA), following CABG surgery for coronary atherosclerotic heart disease in the same hospital. Patients were segregated into a diabetes mellitus (DM) or non-diabetes mellitus (No DM) group, according to whether or not they suffered from DM. All data were reviewed by one cardiac surgeon and two cardiologists, the latter contacting patients for follow-up outcomes by telephone, mail or visit. The study was approved by the Institutional Ethics Committee of Beijing Anzhen Hospital.

### Definitions used in this study

Patients’ data before PCI was defined as baseline data. DM was defined as either a previous diagnosis of DM treated with diet, oral agents, peptide analogs or insulin, or a new diagnosis after index hospitalization [12] before PCI. A graft with a stenosis of > 70% of its diameter was defined as stenosis. A graft with stenosis or occlusion was classified as a diseased graft in this study. The classification of ischemic territory was based on the results of coronary angiography (CAG) after re-hospitalization and also referred to the results prior to CABG. NCA related to ischemic territory was defined as relevant NCA. Paclitaxel-eluting and sirolimus-eluting stents were defined as first-generation DES. Everolimus-eluting and zotarolimus-eluting stents were defined as second-generation DES. Procedural complications refer to complications post PCI. PCI failure was defined as failure to implant a stent at one lesion site.

The primary end-point was a major adverse cardiac event (MACE), defined as the combined incidence of either cardiac death, myocardial infarction (MI), heart failure (HF) or revascularization, as independently adjudicated by an events committee. Cardiac death was defined as any death due to MI, HF, lethal arrhythmia or sudden death in a previously stable patient [13]. MI was defined as: (1) elevation of myocardial enzymes such as cardiac troponin T (cTnT) or creatine kinase-muscle/brain (CK-MB) > 2 fold higher than the upper normal value and (2) changes in ST-segment and T-wave (ST-T) on electrocardiography [14]. HF was defined as hospitalization for progressive heart failure with clinical and radiographic signs. Revascularization was defined as undergoing a subsequent revascularization procedure by PCI or Redo CABG after discharge from the Department of Cardiology, Anzhen Hospital.

### Statistical analysis

All results were analyzed using Stata SE for Windows, version 15.0 (Stata Corporation, College Station, TX, USA) statistical package and IBM SPSS Statistics for Windows, version 22.0. Categorical variables are presented as raw numbers (%) and continuous variables as means ± standard deviation. Comparisons of the DM and No DM groups were accomplished using a Fisher’s exact test for each variable and Mann-Whitney-Wilcox nonparametric test for continuous variables. To reduce the impact of potential confounding on MACEs on the results of the observational study, 1:1 propensity score matching was conducted to choose patients with comparable baseline data. After evaluation of covariates associated clinically and / or statistically with the treatment group and removal of repeatedly defined or collinear variables, including baseline characteristics, risk factors, medical history, clinical conditions at admission and treatment during hospitalization, 36 variables listed in Fig. 2 were included in the propensity score matching model using greedy nearest neighbor matching without replacement and a caliper of 0.02. The absolute standardized difference in variables included for the calculation of propensity score were compared before and after propensity-score matching. The absolute standardized difference cut-off point for the variables included in the calculation was fixed at 10.0%. After matching, Cox proportional hazards regression analysis was also conducted to assess the association between variables and follow-up outcomes. Univariable Cox proportional hazards regression models were initially conducted, followed by multivariable Cox proportional hazards regression models. The candidate variables were potential confounding variables that were either mostly included in the propensity score matching model or reported more than once with an effect on cardiac death or MACEs. After forward stepwise selection with inclusion criteria both set at *P* = 0.2, the variables were eventually included in multivariable Cox proportional hazards regression models of cardiac death and MACEs, respectively. Outcomes were compared using a log-rank test and presented as Kaplan-Meier curves. For all analyses reported, *P* values were 2-sided. Statistical differences were considered significant for values of *P* <0.05.

## Results

### Baseline characteristics

**Fig. 1:**
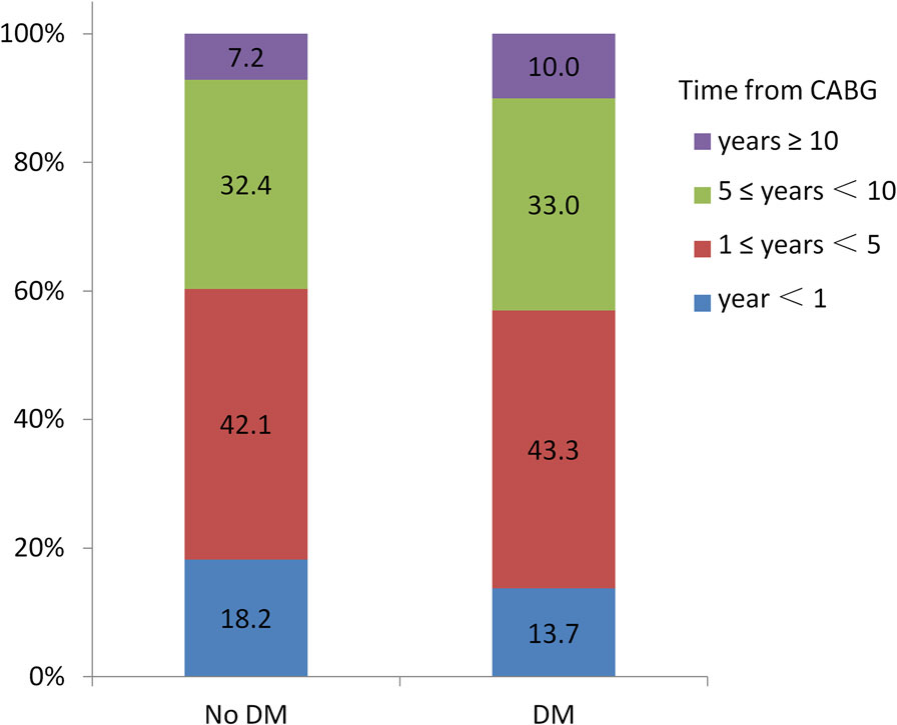
Distribution of patients accepting subsequent PCI with DESs by time period after coronary artery bypass graft surgery, *P* = 0.261. PCI = percutaneous coronary intervention, DESs = drug-eluting stents

**Fig. 2:**
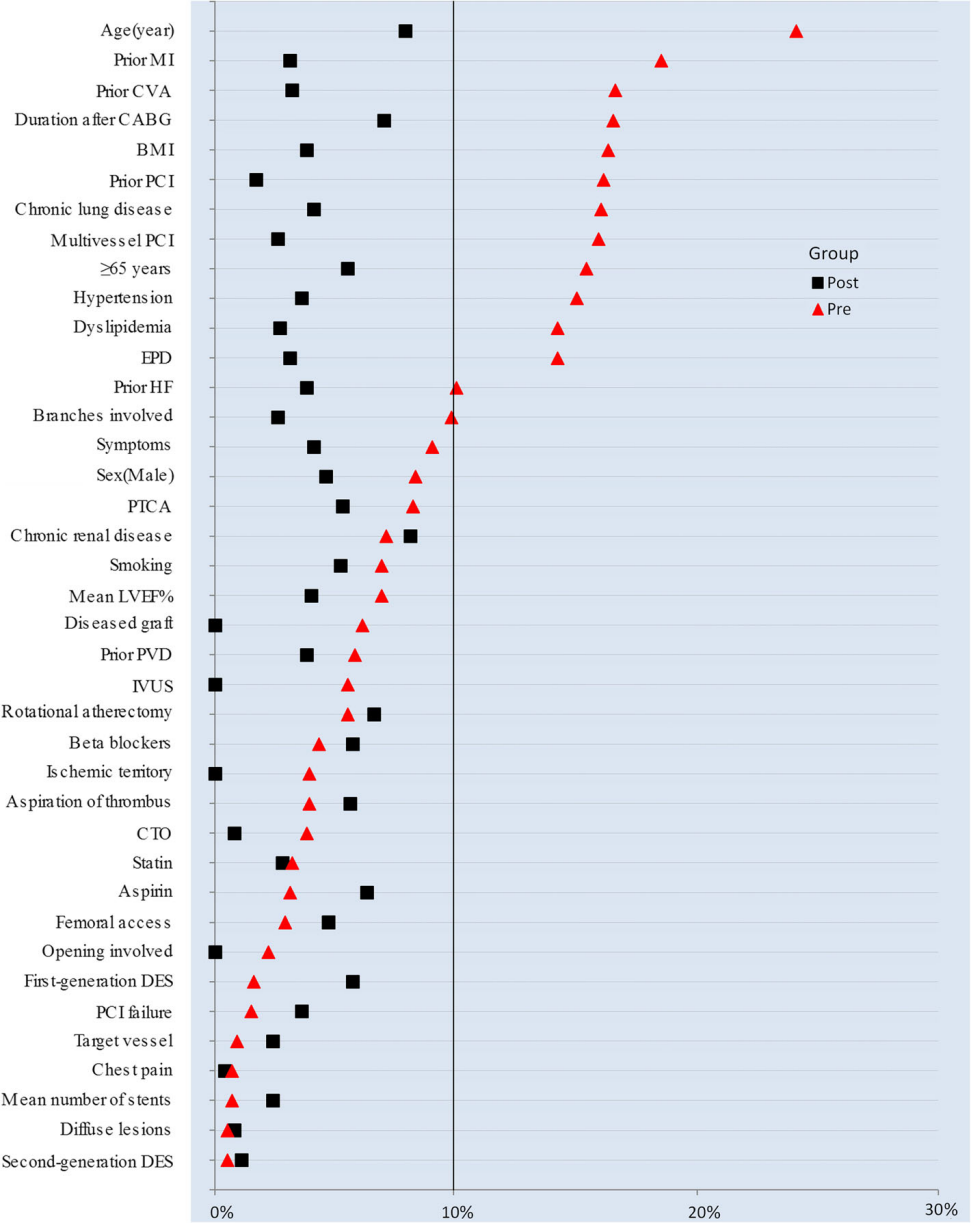
Dotplot of absolute standardized differences before and after matching

In this study, 724 patients were included of which 351 patients (48.5%) exhibited DM. In the DM group, 43.3% of patients presented between 1 and 5 years after CABG and 33.0% between 5 and 10 years, as shown in Fig. 1. The absolute standardized difference values before and after matching are shown in Fig. 2. Following the matching, absolute standardized differences < 10.0% for those variables included indicated a relatively small imbalance.

The baseline characteristics are shown in Table 1. After matching, there were 256 patients in each group, and the statistical difference in age (years), BMI, duration of period after CABG, numbers of patients that were >65 years old, had chronic lung disease, prior cerebrovascular accidents (CVA) or prior PCI were not as significant as they had been before matching the two groups. The characteristics of the CAGs of the diseased grafts and relevant NCAs are also shown in Table 1, with no significant difference between the two groups before matching. The relevant indicators of blood sugar of patients such as glucose concentratrion and glycosylated hemoglobin (HbA1c) are also shown in Table 1.

**Table 1:** Comparison of baseline characteristics of non-diabetic vs diabetic patients with prior CABG (*n* = 724)

Variable	Unmatched			Matched		
	No DM	DM	*P* value	No DM	DM	*P* value
	*n* = 373	*n* = 351		*n* = 256	*n* = 256	
Demographics						
Age (year)	61.52 ± 9.23	63.64 ± 8.15	0.001	62.25 ± 8.71	62.86 ± 8.13	0.409
≥ 65 years	146(39.1%)	165(47.0%)	0.036	101(39.5%)	108(42.2%)	0.590
Sex (Male)	290(77.7%)	261(74.4%)	0.296	192(75.0%)	197(77.0%)	0.679
Comorbidities						
Hypertension	263(70.5%)	268(76.4%)	0.078	188(73.4%)	192(75.0%)	0.762
Dyslipidemia	167(44.8%)	182(51.9%)	0.063	131(51.2%)	127(49.6%))	0.791
Chronic renal disease	12(3.2%)	16(4.6%)	0.441	10(3.9%)	6(2.3%)	0.447
Chronic lung disease	20(5.4%)	8(2.3%)	0.035	10(3.9%)	8(3.1%)	0.811
Prior PVD	43(11.5%)	34(9.7%)	0.470	24(9.4%)	27(10.5%)	0.768
Prior CVA	49(13.1%)	66(18.8%)	0.042	40(15.6%)	37(14.5%)	0.805
Prior MI	52(13.9%)	75(21.4%)	0.011	43(16.8%)	40(15.6%)	0.811
Prior HF	6(1.6%)	2(0.6%)	0.288	3(1.2%)	2(0.8%)	1.000
Prior PCI	14(3.8%)	26(7.4%)	0.035	13(5.1%)	14(5.5%)	1.000
Smoking	238(63.8%)	212(60.4%)	0.358	162(63.3%)	167(65.2%)	0.712
BMI	25.70 ± 3.00	26.19 ± 3.18	0.037	25.97 ± 2.98	25.96 ± 3.23	0.965
HbA1c(%)	6.02 ± 1.08	7.50 ± 1.68	<0.001	5.97 ± 1.01	7.48 ± 1.37	<0.001
Blood sugar (mmol/L)	5.59 ± 1.46	7.89 ± 3.04	<0.001	5.43 ± 0.83	7.86 ± 2.77	<0.001
Symptoms			0.245			0.811
Chest pain	356(95.4%)	341(97.2%)	0.456	248(96.9%)	246(96.1%)	0.966
SA	142(39.9%)	126(37.0%)		98(39.5%)	96(39.0%)	
UA	133(37.4%)	124(36.4%)		90(36.3%)	92(37.4%)	
AMI	81(22.8%)	91(26.7%)		60(24.2%)	58(23.6%)	
Others	17(4.6%)	10(2.8%)		8(3.1%)	10(3.9%)	
Mean LVEF%	59.13 ± 9.16	58.46 ± 9.29	0.364	59.06 ± 8.96	58.65 ± 9.03	0.631
Duration after CABG	4.48 ± 3.43	5.03 ± 3.66	0.034	4.68 ± 3.43	4.71 ± 3.52	0.917
CAG characteristics						
Diseased graft	303(81.2%)	278(79.2%)	0.514	207(80.9%)	207(80.9%)	1.000
Relevant NCAs						
CTO	194(52.0%)	176(50.1%)	0.655	128(50.0%)	127(49.6%)	1.000
Diffuse lesions	56(15.0%)	53(15.1%)	1.000	38(14.8%)	37(14.5%)	1.000
Branches involved	104(27.9%)	114(32.5%)	0.195	70(27.3%)	73(28.5%)	0.844
Opening involved	117(31.4%)	106(30.2%)	0.748	81(31.6%)	81(31.6%)	1.000
Ischemic territory			0.885			0.649
One territory	142(38.1%)	137(39.0%)		102(39.8%)	99(38.7%)	
Two territories	172(46.1%)	163(46.4%)		125(48.8%)	121 (47.3%)	
Three territories	59(15.8%)	51(14.5%)		29(11.3%)	36(14.1%)	
SYNTAX Score I	42.0 ± 13.0	43.4 ± 12.7	0.145	42.1 ± 13.1	42.8 ± 12.9	0.575

*AMI* Acute myocardial infarction, *BMI* Body mass index, *CABG* Coronary artery bypass graft, *CTO* Chronic total occlusion, *CVA* Cerebrovascular accident, *DM* Diabetes mellitus, *HbA1c* Glycosylated hemoglobin, *HF* Heart failure, *LVEF* Left ventricular ejection fraction, *NCA* Native coronary artery, *PCI* Percutaneous coronary intervention, *PVD* Peripheral vascular disease, *SA* Stable angina, *SVG* Saphenous vein graft, *UA* Unstable angina

### PCI-related baseline characteristics

Table 2 displays the procedural baseline characteristics, which are also included in the propensity score matching. After matching, PCI was mostly performed in NCA only (No DM: 87.1% vs DM: 87.9%) with first-generation DES used widely (No DM: 62.1% vs DM: 64.8%) in each group. A small proportion of patients also underwent percutaneous coronary angioplasty (PTCA) in addition to stent implantation (No DM: 7.4% vs DM: 9.0%) and PCI failure in stent implantation in one lesion site (No DM: 4.3% vs DM: 5.1%). EPD, rotational atherectomy, aspiration of thrombus or intravascular ultrasound (IVUS) were not greatly used in either group.

**Table 2:** Procedural baseline characteristics of patients with prior CABG (*n* = 724)

Variable	Unmatched			Matched		
	No DM	DM	*P* value	No DM	DM	*P* value
	*n* = 373	*n* = 351		*n* = 256	*n* = 256	
Femoral access	201(53.9%)	185(52.7%)	0.766	138(53.9%)	132(51.6%)	0.658
Target vessel			1.000			0.894
NCA only	327(87.7%)	308(87.7%)		223(87.1%)	225(87.9%)	
NCA and Graft	46(12.3%)	43(12.3%)		35(12.9%)	32(12.1%)	
Multi-vessel PCI	122(32.7%)	90(25.6%)	0.041	73(28.5%)	70(27.3%)	0.844
Stent						
Mean number of stents	1.88 ± 1.13	1.87 ± 1.09	0.874	1.83 ± 1.11	1.89 ± 1.12	0.558
First-generation DES	236(63.3%)	220(62.7%)	0.878	159(62.1%)	166(64.8%)	0.582
Second-generation DES	159(42.6%)	147(41.9%)	0.880	107(41.8%)	106(41.4%)	1.000
PTCA	40(10.7%)	30(8.5%)	0.379	19(7.4%)	23(9.0%)	0.629
PCI failure	18(4.8%)	19(5.4%)	0.739	11(4.3%)	12(5.1%)	0.835
EPD	12(3.2%)	5(1.4%)	0.142	5(2.0%)	4(1.6%)	1.000
Rotational atherectomy	4(1.1%)	6(1.7%)	0.535	4(1.6%)	4(1.6%)	1.000
Aspiration of thrombus	2(0.5%)	3(0.9%)	0.678	1(0.4%)	1(0.4%)	1.000
IVUS	4(1.1%)	6(1.7%)	0.535	3(1.2%)	1(0.4%)	0.624
Medication						
Aspirin	368(98.7%)	345(98.3%))	0.767	253(98.8%)	252(98.4%)	0.725
Statin	325(87.1%)	300(85.5%)	0.519	200(78.1%)	203(79.3%)	0.829
Beta blockers	289(77.5%)	277(78.9%)	0.654	220(85.9%)	221(86.3%)	1.000

*CABG* Coronary artery bypass graft, *DES* Drug-eluting stent, *DM* Diabetes mellitus, *EPD* Embolic protection devices, *IVUS* Intravascular ultrasound, *NCA* Native coronary artery, *PCI* Percutaneous coronary intervention, *PTCA* Percutaneous coronary angioplasty

### Procedural complications

Table 3 describes recorded procedural complications. After matching, the DM group exhibited slightly high in-hospital mortality, with a higher incidence of angina after 24 h (5.1%), periprocedural MI (1.2%), stroke (0.8%) and bleeding (1.6%), but there was no significant difference between the two groups.

**Table 3:** Procedure-related complications of patients with prior CABG (*n* = 724)

Outcomes	Unmatched			Matched		
	No DM	DM	*P* value	No DM	DM	*P* value
	*n* = 373	*n* = 351		*n* = 256	*n* = 256	
In-hospital mortality	0(0.0%)	2(0.6%)	0.235	0(0.0%)	1(0.4%)	1.000
Procedural complications						
Dysrhythmia	1(0.3%)	2(0.6%)	0.613	1(0.4%)	1(0.4%)	1.000
Angina in 24 h	13(3.5%)	22(6.3%)	0.086	6(2.3%)	13(5.1%)	0.159
Periprocedural MI	4(1.1%)	5(1.4%)	0.746	2(0.8%)	3(1.2%)	1.000
AHF	1(0.3%)	3(0.9%)	0.359	1(0.4%)	1(0.4%)	1.000
Stroke	1(0.3%)	2(0.6%)	0.613	0(0.0%)	2(0.8%)	0.499
Dissection	1(0.3%)	2(0.6%)	0.613	1(0.4%)	1(0.4%)	1.000
Acute closure	0(0.0%)	2(0.6%)	0.235	0(0.0%)	1(0.4%)	1.000
Bleeding	2(0.5%)	5(1.4%)	0.273	2(0.8%)	4(1.6%)	0.686

*AHF* Acute heart failure, *CABG* Coronary artery bypass graft, *DM* Diabetes mellitus, *MI* Myocardial infarction, *PCI* Percutaneous coronary intervention

### Follow-up outcomes

**Fig. 3:**
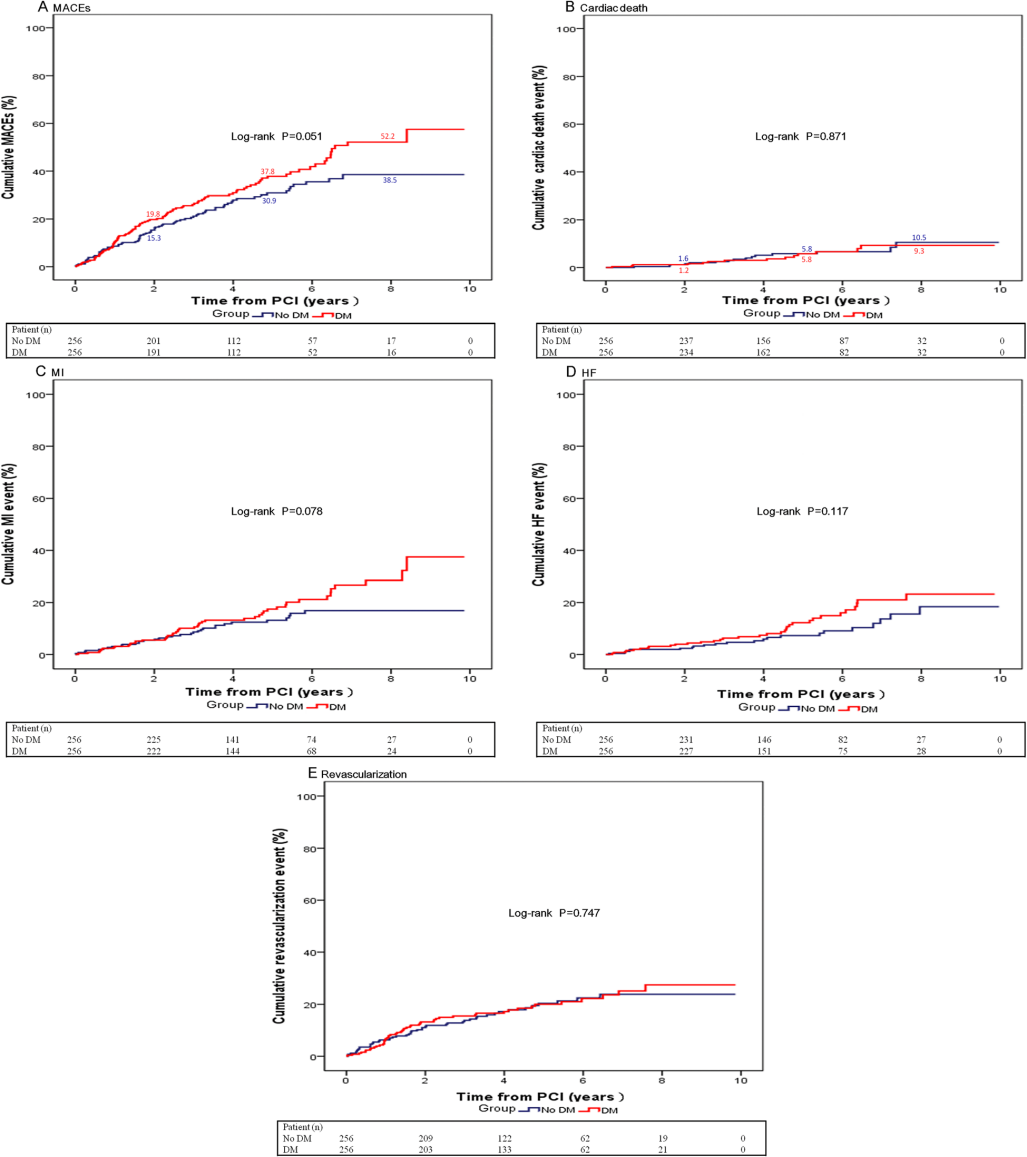
Incidence of MACEs (**a**), cardiac death (**b**), MI (**c**), HF (**d**) or revascularization (**e**) in the No DM group (blue line) compared with the DM group (red line) using the Kaplan-Meier method. *P* value was calculated by log-rank test. DM = diabetes mellitus, HF = acute heart failure, MACEs = major adverse cardiac events, MI = myocardial infarction

**Fig. 4:**
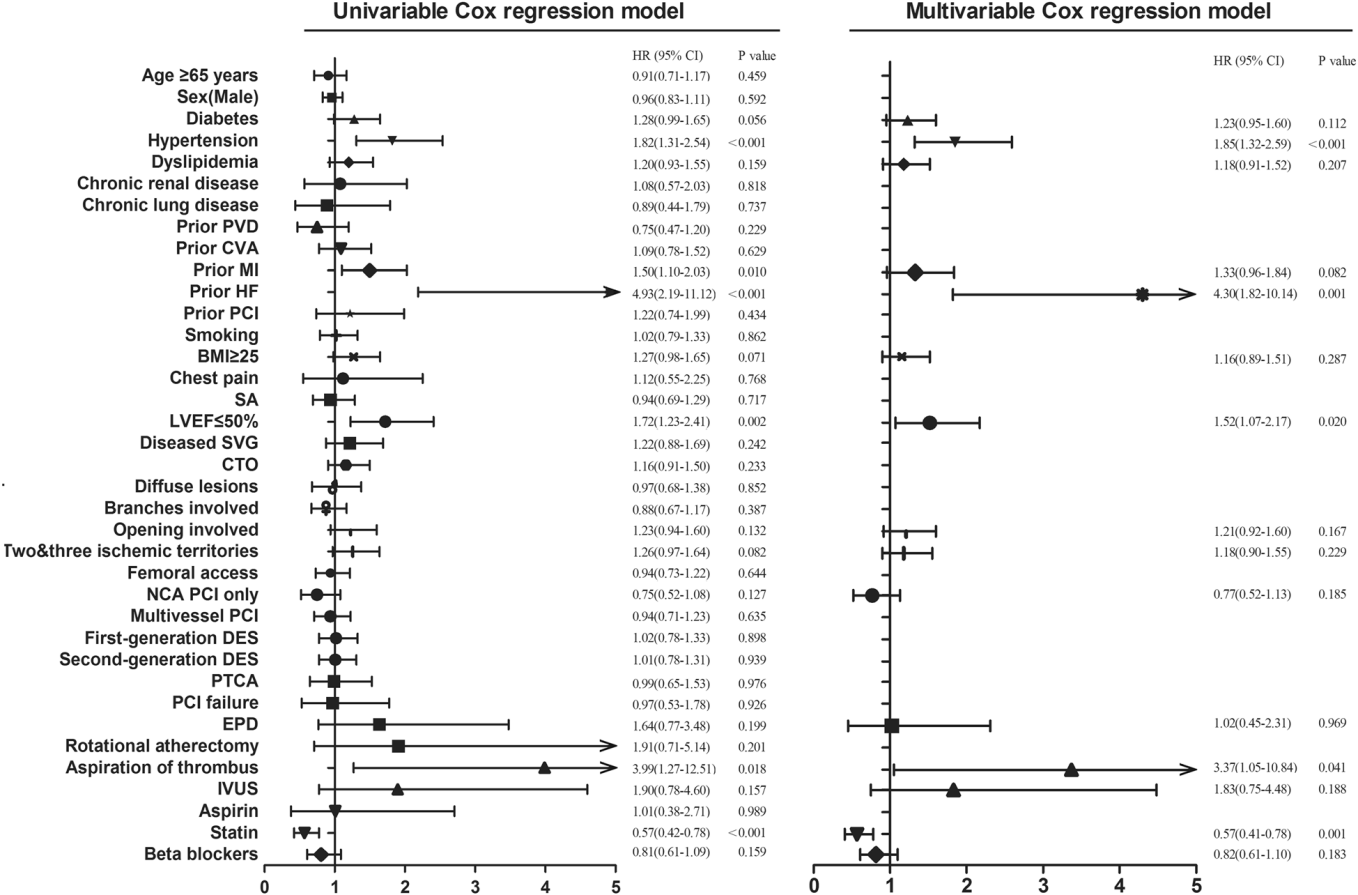
Univariable and multivariable analyses of factors associated with major adverse cardiac events (MACEs) are performed with Cox proportional hazards regression, HRs with 95% CIs are shown. Abbreviations as in Tables 1 and 2

Complete follow-up data were obtained in the overall study population, as displayed in Table 4 and Fig. 3. The median follow-up duration was 5.13 years. After matching, Kaplan-Meier curves (Fig. 4a) indicated that the cumulative overall rate of MACEs was higher in the DM group at 2 years (No DM: 15.3% vs DM: 19.8%), 5 years (No DM: 30.9% vs DM: 37.8%) and at 8 years (No DM: 38.5% vs DM: 52.2%) (hazard ratio [HR]: 1.35; 95% confidence interval [CI]: 1.00 to 1.83 for DM vs No DM; *P* = 0.052), with an apparent increase in the difference between the two groups over time. The curves indicated that two-, 5- and 8-year incidence of cardiac death in each group (Fig. 4b) were similar (No DM: 1.6, 5.8, 10.5% vs DM 1.2, 5.8, 9.3%; HR: 0.94; 95% CI: 0.45 to 1.95 for DM vs No DM; *P* = 0.871). There was also no statistical difference in incidence of MI (HR: 1.49; 95% CI: 0.95 to 2.32 for DM vs No DM; *P* = 0.080), HF (HR: 1.54; 95% CI: 0.90 to 2.63 for DM vs No DM; *P* = 0.120) or repeated revascularization (HR: 1.07; 95% CI: 0.72 to 1.59 for DM vs No DM; *P* = 0.747) between the two groups, although there was a trend that the incidence of MI or HF in the DM group increased over time.

**Table 4:** Follow-up outcomes of patients with prior CABG (*n* = 724)

Outcomes	Unmatched			Matched		
	No DM	DM	*P* value	No DM	DM	*P* value
	*n* = 373	*n* = 351		*n* = 256	*n* = 256	
MACEs	114(30.6%)	128(36.5%)	0.055	75(29.3%)	96(37.5%)	0.051
Cardiac death	18(4.8%)	18(5.1%)	0.695	15(5.9%)	14(5.5%)	0.871
MI	43(11.5%)	62(17.7%)	0.010	33(12.9%)	47(18.4%)	0.078
HF	39(10.5%)	48(13.7%)	0.095	22(8.6%)	33(12.9%)	0.117
Revascularization	66(17.7%)	66(18.8%)	0.593	47(18.4%)	50(19.5%)	0.747

*CABG* Coronary artery bypass graft, *DM* Diabetes mellitus, *HF* Acute heart failure, *MACEs* Major adverse cardiac events, *MI* Myocardial infarction

Multivariable Cox proportional hazards regression (Fig. 4) demonstrated that patients with hypertension (adjusted HR: 1.85; 95% CI: 1.32 to 2.59; P<0.001), aspiration of thrombus during PCI (adjusted HR: 3.37; 95% CI: 1.05 to 10.84; *P* = 0.041), prior HF (adjusted HR: 4.30; 95% CI: 1.82 to 10.14; *P* = 0.001) or LVEF<50% (adjusted HR: 1.52; 95% CI: 1.07 to 2.17; *P* = 0.020) were more likely to suffer a MACE. Additionally, use of a statin provided protection from MACEs (adjusted HR: 0.57; 95% CI: 0.41 to 0.78; *P* = 0.001).

### Subgroup analysis of PCI in different target vessels

To reduce the influence of PCI in different target vessels, we performed two additional subgroup analyses of patients that had undergone PCI only in the NCA or PCI in both the NCA and graft. The baseline characteristics of the two subgroups are detailed within Additional file 1: Tables S1, S2 and S3. The follow-up outcomes of the two subgroups are provided separately in Tables S4, S5 and Figures S1, S2.

The follow-up outcomes of patients that underwent PCI in only the NCA were consistent with all clinical endpoints experienced by the whole population. Compared with the non-diabetic patients who had previously undergone CABG, subsequent PCI in only the NCA in diabetic patients appeared to result in similar outcomes (Table S4, Figure S1), including MACEs (adjusted HR: 1.13; 95% CI: 0.85 to 1.49 for DM vs No DM; *P* = 0.325), cardiac death (adjusted HR: 0.85; 95% CI: 0.41 to 1.78 for DM vs No DM; *P* = 0.781), MI (adjusted HR: 1.32; 95% CI: 0.84 to 2.01 for DM vs No DM; *P* = 0.069), HF (adjusted HR: 1.41; 95% CI: 0.87 to 2.27 for DM vs No DM; *P* = 0.211) or repeated revascularization (adjusted HR: 0.93; 95% CI: 0.64 to 1.37 for DM vs No DM; *P* = 0.836).

When it came to the follow-up outcomes of patients with PCI in both the NCA and graft (Table S5, Figure S2), diabetic patients with subsequent PCI in both the NCA and graft were more likely to experience MACEs (adjusted HR: 4.00; 95% CI: 1.67 to 9.58 for DM vs No DM; *P* = 0.003), cardiac death (adjusted HR: 16.04; 95% CI: 1.58 to 162.50 for DM vs No DM; *P* = 0.066), MI (adjusted HR: 3.78; 95% CI: 1.29 to 11.06 for DM vs No DM; *P* = 0.010), HF (adjusted HR: 7.61; 95% CI: 1.06 to 54.57 for DM vs No DM; *P* = 0.091) or repeated revascularization (adjusted HR: 3.36; 95% CI: 1.05 to 10.68 for DM vs No DM; *P* = 0.276).

### Subgroup analysis of PCI with different generation DESs

To reduce the influence of PCI with different generation DESs, we performed two additional subgroup analyses of patients that had undergone PCI with first-generation DESs or second-generation DESs. The baseline characteristics of the two subgroups are detailed within Additional file 1: Tables S6, S7 and S8. The follow-up outcomes of the two subgroups are provided separately in Tables S9, S10 and Figures S3, S4.

The follow-up outcomes of patients that underwent PCI with first-generation DESs were consistent with all clinical endpoints experienced by the whole population. Compared with the non-diabetic patients who had previously undergone CABG, subsequent PCI with first-generation DESs in diabetic patients appeared to result in similar outcomes (Table S9, Figure S3).

When it came to the follow-up outcomes of patients with PCI with second-generation DESs (Table S10, Figure S4), diabetic patients with subsequent PCI with second-generation DESs were more likely to experience MACEs (adjusted HR: 1.76; 95% CI: 1.00 to 3.08 for DM vs No DM; *P* = 0.016), MI (adjusted HR: 1.90; 95% CI: 0.75 to 4.81 for DM vs No DM; *P* = 0.038) and HF (adjusted HR: 1.87; 95% CI: 0.72 to 4.89 for DM vs No DM; *P* = 0.018). The outcomes of cardiac death (adjusted HR: 1.10; 95% CI: 0.01 to 1.47 for DM vs No DM; *P* = 0.852) and repeated revascularization (adjusted HR: 1.67; 95% CI: 0.79 to 3.53 for DM vs No DM; *P* = 0.305) are similar between two groups.

### Follow-up outcomes of patients with incomplete revascularization vs complete revascularization

We reviewed the coronary angiography files, 37 patients (5.1%) received incomplete revascularization (IR) by PCI and 687 patients (94.9%) received complete revascularization (CR) by PCI after CABG. Follow-up outcomes of patients with IR vs CR are shown in Table S11. Compared with patients with CR, patients with IR are more likely to have MACEs (40.5% vs 33.0%), cardiac death (8.1% vs 4.8%) and MI (21.6% vs 14.1%), though there are no significant differences between two groups.

## Discussion

We performed a retrospective observational study to explore the outcomes of PCI with DES in diabetic vs non-diabetic patients who had previously undergone CABG in our single-center registry. We found that, compared to non-diabetic patients with prior CABG, subsequent PCI within the NCA with DES in diabetic patients appeared to result in a similar overall incidence of MACEs, cardiac death, MI, HF or repeated revascularization, extending our current understanding of the safety and efficacy of DES even in high-risk patients with prior CABG. This suggests that a DES may be considered the default option in these patient populations. In this study we also found that hypertension, prior HF, LVEF<50% and aspiration of thrombus are predictive of overall MACEs and patients taking statins are less likely to experience MACEs. Our results were based on matching propensity scores, which suggests that our findings are not due to negative confounding.

Diabetic patients with CAD are reported to have dysfunctional endothelial cells, increased atherosclerotic burden and fragile lipid-rich plaques [15, 16], microcirculation disorder involving smaller vessels, and prothrombotic and proinflammatory states [17, 18], which are related to progression of NCA disease. It is confirmed that CAD in diabetic patients appears as diffuse atherosclerosis with chronic total occlusion (CTO), opening and bifurcation lesions or multivessel disease and left main disease [19], leading to fewer amenable options for re-intervention and suboptimal stent expansion [8]. In this study, although the characteristics of the lesions in the NCAs relevant to ischemic territory are similar in both diabetic and non-diabetic patients, those in each group represent a high proportion of the CTO lesions, openings involving lesions, branches involving lesions or diffuse lesions. We consider that this is due to the combined CAD risk factors, such as hypertension, dyslipidemia, diabetes mellitus, fat, smoking and gender. In order to remove the influence of confounding CAD risk factors and to compare between the DM group and No DM group more precisely, we used the propensity score matching method, described in detail in the statistical analysis section.

Atherosclerosis is also reported to play an important role in later graft failure (graft age >6 months) [8]. Graft atherosclerosis in diabetic patients has a larger necrotic core with unstable plaques [20], which is friable and more prone to distal coronary embolization [2]. Compared with non-diabetic patients with prior CABG, diabetic patients have a higher rate of graft stenosis and recurrent myocardial ischemic events [21, 22], due to the progression of NCA disease or graft failure [2]. In this study the majority of patients in each group had diseased grafts (81.2 and 79.2% in the No DM and DM groups, respectively), and a small proportion of the remaining patients in each group had myocardial ischema caused by isolated NCA lesions as a consequence of the progression of NCA disease (18.8 and 20.8% in the No DM and DM groups, respectively).

DES are superior to bare metal stents (BMS), in terms of their strut thickness and polymer coating composition, reducing repeat revascularization and in-stent thrombosis in addition to MI in non-diabetic patients [19]. Published literature indicates that PCI with DES in diabetic patients compared with non-diabetic patients results in significantly higher mortality, reinfarction, and repeat revascularization for in-stent restenosis [23–25]. The pathological mechanism of in-stent restenosis in diabetic patients is associated with excessive endothelial hyperplasia, vascular remodeling or increased platelet aggregation [12]. However, in this study we found different results, especially when performing additional subgroup analysis of patients with PCI in only the NCA, in that PCI in the NCA with DES in diabetic patients compared with non-diabetic patients did not result in a high incidence of cardiac death, HF or repeat revascularization, and the incidence of MI between the two groups was not significantly different. However, one key observation should be clearly noted, that patients in that study included those without prior CABG. Conversely, all patients included in this study underwent prior CABG in our cardiac center, which could be considered a pretreatment for diabetic patients and functions as protection. In addition, all were high risk patients, especially in the DM group. They were older with a greater number of comorbidities and had severe NCA or graft lesions.

The studies of Ahmed [26] and Ashfaq [27] reported the influence of DM on outcomes in saphenous vein graft (SVG) stenting, with similar conclusions, that PCI with DES in diabetics resulted in long-term overall rates of MACEs, death, MI and repeat revascularization that were worse than in non-diabetics, quite different from Pendyala’s conclusion that diabetic patients undergoing SVG PCI had similar long-term outcomes [12]. In the present study, after analysis of the whole study population (after matching, *n* = 512), we found that the overall incidence of MACEs (DM: 37.5% vs No DM: 29.3%), principally driven by MI (DM: 18.4% vs No DM: 12.9%), were not statistically different between the two groups despite an increasing trend over time. Considering the conflicting data of all the patients that received PCI in the NCA and that a minority of patients were treated with PCI in both graft and NCA (No DM: 12.9% vs DM: 12.1), we performed further subgroup analysis of patients with PCI in only the NCA (*n* = 635). We found that, compared to non-diabetic patients with prior CABG, subsequent DES in only the NCA of diabetic patients appeared to result in similar outcomes, such as rates of MACEs, cardiac death, MI, HF and repeat revascularization. Additional subgroup analysis of patients with PCI in both the NCA and graft, despite the small sample size of patients in this subgroup (*n* = 89), demonstrated that, compared to non-diabetic patients with prior CABG, subsequent DES in the NCA and grafts of diabetic patients resulted in worse outcomes, consistent with Ahmed and Ashfaq’s studies. According to the 2018 ESC/EACTS Guidelines for myocardial revascularization [2], it is recommended that PCI in the NCA should be considered rather than PCI in an SVG graft, because that is associated with a high risk of periprocedural MI [28] and worse long-term outcomes such as all-cause death, MI or revascularization [29] for no-reflow, subsequent in-stent restenosis, distant target lesions and excessive tortuosity [8], especially for PCI in an SVG of a diabetic patient for graft atherosclerosis with a larger necrotic core and friable plaques [20].

In this study, we also provided follow-up outcomes of patients with IR vs CR by PCI. Achieving CR of all significantly obstructed coronary artery has been an established goal of PCI, and more recent data demonstrate a salutary effect of CR following PCI on long-term outcomes. IR is associated with increased mortality following PCI, as well as with an increased incidence of MI, repeat revascularization, and MACCEs [30]. Though, in our study the sample size of patients with IR was too small (5.1%), which would influence statistical results, we still believed that it made a little sense, compared with patients with CR, patients with IR were more likely to have MACEs (40.5%), cardiac death (8.1%) and MI (21.6%), though there were no significant difference between two groups. Further randomized controlled trial study with a larger sample size and longer follow-up may be required for patient with prior CABG.

### Limitations

Firstly, this was a retrospective observational single-center study and so is subject to all the limitations of observational single-center studies, such as patient selection and a single therapeutic method, which might affect the results. Secondly, the angiography film results were analyzed by one cardiac surgeon and one cardiologist. Thirdly, the classification of graft lesions was in reference to the evaluation criteria of native vessels. Fourthly, the decision to perform PCI for each patient was taken by 2 operators, mostly based on an evaluation of the CAG results. Fifthly, 6 non-DM patients who had diabetes during the follow-up period were excluded from this study. Sixthly, we didn’t do PS matching for sub-group analysis. Despite these limitations, the results were derived from the largest angiographic study in patients with prior CABG so far published. In addition, the statistical analyses utilized rigorous methodology.

## Conclusions

Compared to non-diabetic patients with prior CABG, subsequent PCI in an NCA with DES in diabetic patients appears to result in a similar incidence of overall MACEs, cardiac death, MI, HF and repeated revascularization, suggesting that DES may be considered the default option for these patient populations. We also found that hypertension, prior HF, LVEF<50% and aspiration of thrombus are predictive for overall rate of MACE in diabetic patients with prior CABG. Patients that were administered statins were less likely to experience MACEs.

## Supplementary information

**Additional file 1: Table S1.** Comparison of baseline characteristics of two subgroups of patients with PCI in different target vessels. **Table S2.** Procedural baseline characteristics of two subgroups of patients with PCI in different target vessels. **Table S3.** Procedure-related complications of two subgroups of patients with PCI in different target vessels. **Table S4.** Follow-up outcomes of subgroup of patients with PCI in NCA only. **Table S5.** Follow-up outcomes of subgroup of patients with PCI in NCA and Graft. **Table S6.** Comparison of baseline characteristics of two subgroups of patients with PCI with different generation DESs. **Table S7.** Procedural baseline characteristics of two subgroups of patients with PCI with different generation DESs. **Table S8.** Procedure-related complications of two subgroups of patients with PCI with different generation DESs. **Table S9.** Follow-up outcomes of patients with first-generation DESs PCI. **Table S10.** Follow-up outcomes of patients with second-generation DESs PCI. **Table S11.** Follow-up outcomes of patients with incomplete revascularization vs complete revascularization.

**Additional file 2: Figure S1**. Incidence of MACEs (A), cardiac death (B), MI(C), HF(D) or revascularization (E) of patients with PCI in only NCA (No DM vs DM) using the Kaplan-Meier method. *P* value was calculated by log-rank test. DM = diabetes mellitus, HF = acute heart failure, NCA = native coronary artery, MACEs = major adverse cardiac events, MI = myocardial infarction.

**Additional file 3: Figure S2.** Incidence of MACEs (A), cardiac death (B), MI(C), HF(D) or revascularization (E) of patients with PCI in NCA and graft (No DM vs DM) using the Kaplan-Meier method. *P* value was calculated by log-rank test. DM = diabetes mellitus, HF = acute heart failure, NCA = native coronary artery, MACEs = major adverse cardiac events, MI = myocardial infarction.

**Additional file 4: Figure S3.** Incidence of MACEs (A), cardiac death (B), MI(C), HF(D) or revascularization (E) of patients with first-generation DES PCI (No DM vs DM) using the Kaplan-Meier method. *P* value was calculated by log-rank test. DM = diabetes mellitus, HF = acute heart failure, NCA = native coronary artery, MACEs = major adverse cardiac events, MI = myocardial infarction.

**Additional file 5: Figure S4**. Incidence of MACEs (A), cardiac death (B), MI(C), HF(D) or revascularization (E) of patients with second-generation DES PCI (No DM vs DM) using the Kaplan-Meier method. *P* value was calculated by log-rank test. DM = diabetes mellitus, HF = acute heart failure, NCA = native coronary artery, MACEs = major adverse cardiac events, MI = myocardial infarction.

## Data Availability

The datasets generated and analyzed for this current study are available from the corresponding author upon reasonable request.

## Abbreviations

AHF: Acute heart failure
AMI: Acute myocardial infarction
BMI: Body mass index
CABG: Coronary artery bypass graft
CTO: Chronic total occlusion
CVA: Cerebrovascular accident
DES: Drug-eluting stent
DM: Diabetes mellitus
HbA1c: Glycosylated hemoglobin
HF: Heart failure
LVEF: Left ventricular ejection fraction
NCA: Native coronary artery
MACEs: Major adverse cardiac events
PCI: Percutaneous coronary intervention
PVD: Peripheral vascular disease
SA: Stable angina
SVG: Saphenous vein graft
UA: Unstable angina
