# Supplementary material for: Percutaneous coronary intervention in diabetic versus non-diabetic patients with prior coronary artery bypass grafting: a propensity score matching study
Source: BMC Cardiovasc Disord. 2020 Apr 6;20:159. doi: 10.1186/s12872-020-01447-8 (PMC7137249; doi:10.1186/s12872-020-01447-8)
Supplement: Supplementary file 1 — Additional file 1: Table S1. Comparison of baseline characteristics of two subgroups of patients with PCI in different target vessels. Table S2. Procedural baseline characteristics of two subgroups of patients with PCI in different target vessels. Table S3. Procedure-related complications of two subgroups of patients with PCI in different target vessels. Table S4. Follow-up outcomes of subgroup of patients with PCI in NCA only. Table S5. Follow-up outcomes of subgroup of patients with PCI in NCA and Graft. Table S6. Comparison of baseline characteristics of two subgroups of patients with PCI with different generation DESs. Table S7. Procedural baseline characteristics of two subgroups of patients with PCI with different generation DESs. Table S8. Procedure-related complications of two subgroups of patients with PCI with different generation DESs. Table S9. Follow-up outcomes of patients with first-generation DESs PCI. Table S10. Follow-up outcomes of patients with second-generation DESs PCI. Table S11. Follow-up outcomes of patients with incomplete revascularization vs complete revascularization. [file 12872_2020_1447_MOESM1_ESM.docx]

**Table S1** Comparison of baseline characteristics of two subgroups of patients with PCI in different target vessels

| Variable | PCI in only NCA (n=635) | | |  | PCI in NCA and Graft(n=89) | | |
| --- | --- | --- | --- | --- | --- | --- | --- |
|  | No DM | DM | P value |  | No DM | DM | P value |
|  | n=327 | n=308 |  |  | n=46 | n=43 |  |
| Demographics |  |  |  |  |  |  |  |
| Age(year) | 61.6±8.9 | 63.9±8.1 | 0.001 |  | 61.1±11.2 | 62.1±8.7 | 0.654 |
| ≥65 years | 127(38.8%) | 145(47.1%) | 0.037 |  | 19(41.3%) | 20(46.5%) | 0.673 |
| Sex(Male) | 255(78.0%) | 224(72.7%) | 0.140 |  | 35(76.1%) | 37(86.0%) | 0.286 |
| Comorbidities |  |  |  |  |  |  |  |
| Hypertension | 228(69.7%) | 237(76.9%) | 0.048 |  | 35(76.1%) | 31(72.1%) | 0.809 |
| Dyslipidemia | 141(43.1%) | 161(52.3%) | 0.021 |  | 26(56.5%) | 21(48.8%) | 0.527 |
| Chronic renal disease | 10(3.1%) | 14(4.5%) | 0.406 |  | 2(4.3%) | 2(4.7%) | 1.000 |
| Chronic lung disease | 17(5.2%) | 6(1.9%) | 0.033 |  | 3(6.5%) | 2(4.7%) | 1.000 |
| Prior PVD | 36(11.0%) | 31(10.1%) | 0.796 |  | 7(15.2%) | 3(7.0%) | 0.318 |
| Prior CVA | 41(12.5%) | 61(19.8%) | 0.013 |  | 8(17.4%) | 5(11.6%) | 0.553 |
| Prior MI | 44(13.5%) | 62(20.1%) | 0.026 |  | 8(17.4%) | 13(30.2%) | 0.212 |
| Prior HF | 6(1.8%) | 1(0.3%) | 0.124 |  | 0(0.0%) | 1(2.3%) | 0.483 |
| Prior PCI | 12(3.7%) | 23(7.5%) | 0.038 |  | 2(4.3%) | 3(7.0%) | 0.670 |
| Smoking | 206(63.0%) | 178(57.8%) | 0.194 |  | 32(69.6%) | 34(79.1%) | 0.341 |
| BMI | 25.7±2.9 | 26.1±3.1 | 0.161 |  | 25.6±3.8 | 27.2±3.6 | 0.052 |
| HbA1c(%) | 6.0±1.0 | 7.5±1.7 | ＜0.001 |  | 6.2±1.4 | 7.6±1.4 | ＜0.001 |
| Blood sugar(mmol/L) | 5.6±1.5 | 7.9±3.1 | ＜0.001 |  | 5.6±1.0 | 8.1±2.4 | ＜0.001 |
| Symptoms |  |  | 0.226 |  |  |  | 1.000 |
| Chest pain | 311(95.1%) | 299(97.1%) | 0.624 |  | 45(97.8%) | 42(97.7%) | 0.202 |
| SA | 122(39.2%) | 115(38.5%) |  |  | 20(44.4%) | 11(26.2%) |  |
| UA | 119(38.3%) | 107(35.8%) |  |  | 14(31.1%) | 17(40.5%) |  |
| AMI | 70(22.5%) | 77(25.8%) |  |  | 11(24.4%) | 14(33.3%) |  |
| Others | 16(4.9%) | 9(2.9%) |  |  | 1(2.2%) | 1(2.3%) |  |
| Mean LVEF% | 59.1±9.2 | 58.6±9.3 | 0.550 |  | 59.4±8.9 | 57.3±9.6 | 0.320 |
| Duration after CABG | 4.3±3.3 | 4.8±3.5 | 0.046 |  | 5.7±3.8 | 6.4±4.4 | 0.433 |
| CAG characteristics |  |  |  |  |  |  |  |
| Diseased graft | 257(78.6%) | 235(76.3%) | 0.507 |  | 0(0.0%) | 0(0.0%) | NS |
| Relevant NCAs |  |  |  |  |  |  |  |
| CTO | 159(48.6%) | 146(47.4%) | 0.812 |  | 35(76.1%) | 30(69.8%) | 0.634 |
| Diffuse lesions | 45(13.8%) | 48(15.6%) | 0.575 |  | 11(23.9%) | 5(11.6%) | 0.171 |
| Branches involved | 94(28.7%) | 107(34.7%) | 0.106 |  | 10(21.7%) | 7(16.3%) | 0.595 |
| Opening involved | 100(30.6%) | 91(29.5%) | 0.796 |  | 17(37.0%) | 15(34.9%) | 1.000 |
| Ischemic territory |  |  | 0.938 |  |  |  | 0.083 |
| One territory | 133(40.7%) | 122(39.6%) |  |  | 9(19.6%) | 15(34.9%) |  |
| Two territories | 148(45.3%) | 140(45.5%) |  |  | 24(52.2%) | 23(53.5%) |  |
| Three territories | 46(14.1%) | 46(14.9%) |  |  | 13(28.3%) | 5(11.6%) |  |
| SYNTAX Score | 41.8±12.9 | 43.3±12.9 | 0.128 |  | 43.8±13.8 | 44.0±11.6 | 0.929 |

AMI=acute myocardial infarction; BMI=body mass index; CABG=coronary artery bypass graft; CTO=chronic total occlusion; CVA=cerebrovascular accident; DM= diabetes mellitus; HbA1c= glycosylated hemoglobin; HF=heart failure; LVEF= left ventricular ejection fraction; NCA=native coronary artery; NS=not state; PCI= percutaneous coronary intervention; PVD=peripheral vascular disease; SA=stable angina; SVG= saphenous vein graft ; UA=unstable angina.

**Table S2** Procedural baseline characteristics of two subgroups of patients with PCI in different target vessels

| Variable | PCI in only NCA (n=635) | | |  | PCI in NCA and Graft(n=89) | | |
| --- | --- | --- | --- | --- | --- | --- | --- |
|  | No DM | DM | P value |  | No DM | DM | P value |
|  | n=327 | n=308 |  |  | n=46 | n=43 |  |
| Femoral access | 172(52.6%) | 160(51.9%) | 0.874 |  | 29(63.0%) | 25(58.1%) | 0.669 |
| Multi-vessel PCI | 101(30.9%) | 81(26.3%) | 0.219 |  | 21(45.7%) | 9(20.9%) | 0.024 |
| Stent |  |  |  |  |  |  |  |
| Mean number of stents | 1.9±1.1 | 1.9±1.1 | 0.533 |  | 1.9±1.4 | 1.4±1.0 | 0.058 |
| First-generation DES | 206(63.0%) | 196(63.6%) | 0.870 |  | 30(65.2%) | 24(55.8%) | 0.393 |
| Second-generation DES | 142(43.4%) | 129(41.9%) | 0.748 |  | 17(37.0%) | 18(41.9%) | 0.669 |
| PTCA | 33(10.1%) | 25(8.1%) | 0.411 |  | 7(15.2%) | 5(11.6%) | 0.759 |
| PCI failure | 15(4.6%) | 18(5.8%) | 0.482 |  | 3(6.5%) | 1(2.3%) | 0.617 |
| EPD | 2(0.6%) | 1(0.3%) | 0.522 |  | 10(21.7%) | 4(9.3%) | 0.147 |
| Rotational atherectomy | 4(1.2%) | 5(1.6%) | 0.746 |  | 0(0.0%) | 1(2.3%) | 0.483 |
| Aspiration of thrombus | 2(0.6%) | 3(1.0%) | 0.678 |  | 0(0.0%) | 0(0.0%) | NS |
| IVUS | 3(0.9%) | 4(1.3%) | 0.718 |  | 1(2.2%) | 2(4.7%) | 0.608 |
| Medication |  |  |  |  |  |  |  |
| Aspirin | 323(98.8%) | 303(98.4%) | 0.746 |  | 45(97.8%) | 42(97.7%) | 1.000 |
| Statin | 285(87.2%) | 264(85.7%) | 0.339 |  | 40(87.0%) | 36(83.7%) | 0.768 |
| Beta blockers | 252(77.1%） | 239(77.6%) | 0.925 |  | 37(80.4%) | 38(88.4%) | 0.388 |

CABG=coronary artery bypass graft; DES=drug-eluting stent; DM= diabetes mellitus; EPD=embolic protection devices; IVUS= intravascular ultrasound; NCA= native coronary artery; NS=not state; PCI=percutaneous coronary intervention; PTCA= percutaneous coronary angioplasty.

**Table S3** Procedure-related complications of two subgroups of patients with PCI in different target vessels

| Outcomes | PCI in only NCA (n=635) | | |  | PCI in NCA and Graft(n=89) | | |
| --- | --- | --- | --- | --- | --- | --- | --- |
|  | No DM | DM | P value |  | No DM | DM | P value |
|  | n=327 | n=308 |  |  | n=46 | n=43 |  |
| In-hospital mortality | 0(0.0%) | 0(0.0%) | NS |  | 0(0.0%) | 2(4.7%) | 0.231 |
| Procedural complications |  |  |  |  |  |  |  |
| Dysrhythmia | 0(0.0%) | 2(0.6%) | 0.235 |  | 1(2.2%) | 0(0.0%) | 1.000 |
| Angina in 24h | 9(2.8%) | 18(5.8%) | 0.075 |  | 4(8.7%) | 4(9.7%) | 1.000 |
| Periprocedural MI | 3(0.9%) | 3(1.0%) | 1.000 |  | 1(2.2%) | 2(4.7%) | 0.608 |
| AHF | 1(0.3%) | 2(0.6%) | 0.613 |  | 0(0.0%) | 1(2.3%) | 0.483 |
| Stroke | 1(0.3%) | 1(0.3%) | 1.000 |  | 0(0.0%) | 1(2.3%) | 0.483 |
| Dissection | 1(0.3%) | 1(0.3%) | 1.000 |  | 0(0.0%) | 1(2.3%) | 0.483 |
| Acute closure | 0(0.0%) | 2(0.6%) | 0.235 |  | 0(0.0%) | 0(0.0%) | NS |
| Bleeding | 2(0.6%) | 4(1.3%) | 0.438 |  | 0(0.0%) | 1(2.3%) | 0.483 |

AHF=acute heart failure; CABG=coronary artery bypass graft; DM= diabetes mellitus; MI= myocardial infarction; NS=not state ;PCI=percutaneous coronary intervention.

**Table S4** Follow-up outcomes of subgroup of patients with PCI in NCA only

|  | No DM | DM | P value | Unadjusted HR (95% CI) | Adjusted HR (95% CI) |
| --- | --- | --- | --- | --- | --- |
|  | n=327 | n=308 |  |  |  |
| MACEs | 102(31.2%) | 106(34.4%) | 0.325 | 1.15(0.87-1.51) | 1.13(0.85-1.49) |
| Cardiac death | 16(4.9%) | 13(4.2%) | 0.781 | 0.90(0.43-1.88) | 0.85(0.41-1.78) |
| MI | 36(11.0%) | 48(15.6%) | 0.069 | 1.49(0.97-2.30) | 1.32(0.84-2.01) |
| HF | 35(10.7%) | 41(13.3%) | 0.211 | 1.33(0.85-2.09) | 1.41(0.87-2.27) |
| Revascularization | 57(17.4%) | 55(17.9%) | 0.836 | 1.04(0.72-1.51) | 0.93(0.64-1.37) |

CABG=coronary artery bypass graft; DM= diabetes mellitus; HF=acute heart failure; MACEs= major adverse cardiac events; MI= myocardial infarction.; HR=hazard ratio; CI= conference interval.

**Table S5** Follow-up outcomes of subgroup of patients with PCI in NCA and Graft

|  | No DM | DM | P value | Unadjusted HR (95% CI) | Adjusted HR (95% CI) |
| --- | --- | --- | --- | --- | --- |
|  | n=46 | n=43 |  |  |  |
| MACEs | 12(26.1%) | 22(51.2%) | 0.003 | 2.80(1.37-5.71) | 4.00(1.67-9.58) |
| Cardiac death | 2(4.3%) | 5(11.6%) | 0.066 | 4.22(0.80-22.16) | 16.04(1.58-162.50) |
| MI | 7(15.2%) | 14(32.6%) | 0.010 | 3.17(1.26-7.96) | 3.78(1.29-11.06) |
| HF | 4(8.7%) | 7(16.3%) | 0.091 | 2.80(0.81-9.69) | 7.61(1.06-54.57) |
| Revascularization | 9(19.6%) | 11(25.6%) | 0.276 | 1.63(0.67-3.96) | 3.36(1.05-10.68) |

CABG=coronary artery bypass graft; DM= diabetes mellitus; HF=acute heart failure; MACEs= major adverse cardiac events; MI= myocardial infarction; HR=hazard ratio; CI= conference interval.

**Table S6** Comparison of baseline characteristics of two subgroups of patients with PCI with different generation DESs

| Variable | First-generation DES (n=375) | | |  | Second-generation DES (n=225) | | |
| --- | --- | --- | --- | --- | --- | --- | --- |
|  | No DM | DM | P value |  | No DM | DM | P value |
|  | n=192 | n=183 |  |  | n=115 | n=110 |  |
| Demographics |  |  |  |  |  |  |  |
| Age(year) | 60.9±9.3 | 63.8±7.8 | 0.001 |  | 60.8±9.2 | 63.9±9.0 | 0.013 |
| ≥65 years | 70(36.5%) | 89(48.6%) | 0.021 |  | 40(34.8%) | 54(49.1%) | 0.032 |
| Sex(Male) | 143(74.5%) | 135(73.8%) | 0.906 |  | 97(84.3%) | 83(75.5%) | 0.099 |
| Comorbidities |  |  |  |  |  |  |  |
| Hypertension | 141(73.4%) | 136(74.3%) | 0.907 |  | 79(68.7%) | 87(79.1%) | 0.095 |
| Dyslipidemia | 92(47.9%) | 96(52.5%) | 0.409 |  | 48(41.7%) | 57(51.8%) | 0.143 |
| Chronic renal disease | 6(3.1%) | 13(7.1%) | 0.100 |  | 2(1.7%) | 3(2.7%) | 0.678 |
| Chronic lung disease | 13(6.8%) | 4(2.2%) | 0.045 |  | 6(5.2%) | 3(2.7%) | 0.500 |
| Prior PVD | 19(9.9%) | 16(8.7%) | 0.726 |  | 14(12.2%) | 14(12.7%) | 1.000 |
| Prior CVA | 26(13.5%) | 33(18.0%) | 0.258 |  | 12(10.4%) | 25(22.7%) | 0.019 |
| Prior MI | 25(13.0%) | 37(20.2%) | 0.071 |  | 17(14.8%) | 23(20.9%) | 0.295 |
| Prior HF | 3(1.6%) | 2(1.1%) | 1.000 |  | 1(0.9%) | 0(0.0%) | 1.000 |
| Prior PCI | 2(1.0%) | 11(6.0%) | 0.010 |  | 5(4.3%) | 9(8.2%) | 0.277 |
| Smoking | 117(60.9%) | 112(61.2%) | 1.000 |  | 88(76.5%) | 64(58.2%) | 0.004 |
| BMI | 26.1±3.0 | 26.3±3.2 | 0.430 |  | 26.0±3.1 | 26.1±3.0 | 0.835 |
| HbA1c(%) | 6.0±1.1 | 7.4±1.7 | ＜0.001 |  | 6.0±0.9 | 7.7±1.8 | ＜0.001 |
| Blood sugar(mmol/L) | 5.6±1.8 | 7.9±3.4 | ＜0.001 |  | 5.5±1.0 | 7.9±2.4 | ＜0.001 |
| Symptoms |  |  | 1.000 |  |  |  | 0.216 |
| Chest pain | 186(96.9%) | 177(96.7%) | 0.693 |  | 107(93.0%) | 107(97.3%) | 0.171 |
| SA | 66(35.5%) | 65(36.7%) |  |  | 46(43.0%) | 33(30.8%) |  |
| UA | 77(41.4%) | 66(37.3%) |  |  | 39(36.4%) | 45(42.1%) |  |
| AMI | 43(23.1%) | 46(26.0%) |  |  | 22(20.6%) | 29(27.1%) |  |
| Others | 6(3.1%) | 6(3.3%) |  |  | 8(7.0%) | 3(2.7%) |  |
| Mean LVEF% | 59.3±9.4 | 58.2±9.2 | 0.281 |  | 58.2±9.2 | 58.9±9.5 | 0.610 |
| Duration after CABG | 4.6±3.4 | 4.8±3.5 | 0.737 |  | 4.1±3.3 | 5.1±3.8 | 0.039 |
| CAG characteristics |  |  |  |  |  |  |  |
| Diseased graft | 154(80.2%) | 147(80.3%) | 1.000 |  | 19(16.5%) | 22(20.0%) | 0.605 |
| Relevant NCAs |  |  |  |  |  |  |  |
| CTO | 93(48.4%) | 89(48.6%) | 1.000 |  | 68(59.1%) | 55(50.0%) | 0.182 |
| Diffuse lesions | 30(15.6%) | 24(13.1%) | 0.557 |  | 16(13.9%) | 19(17.3%) | 0.582 |
| Branches involved | 49(25.5%) | 64(35.0%) | 0.056 |  | 30(26.1%) | 34(30.9%) | 0.462 |
| Opening involved | 55(28.6%) | 54(29.5%) | 0.910 |  | 39(33.9%) | 38(34.5%) | 1.000 |
| Ischemic territory |  |  | 0.881 |  |  |  | 0.285 |
| One territory | 76(39.6%) | 72(39.3%) |  |  | 38(33.0%) | 45(40.9%) |  |
| Two territories | 87(45.3%) | 80(43.7%) |  |  | 56(48.7%) | 52(47.3%) |  |
| Three territories | 29(15.1%) | 31(16.9%) |  |  | 21(18.3%) | 13(11.8%) |  |
| SYNTAX Score | 41.7±13.2 | 42.4±12.0 | 0.601 |  | 41.9±12.5 | 45.0±12.3 | 0.059 |

AMI=acute myocardial infarction; BMI=body mass index; CABG=coronary artery bypass graft; CTO=chronic total occlusion; CVA=cerebrovascular accident; DES= drug-eluting stent; DM= diabetes mellitus; HbA1c= glycosylated hemoglobin; HF=heart failure; LVEF= left ventricular ejection fraction; NCA=native coronary artery PCI= percutaneous coronary intervention; PVD=peripheral vascular disease; SA=stable angina; SVG= saphenous vein graft ; UA=unstable angina.

**Table S7** Procedural baseline characteristics of two subgroups of patients with PCI with different generation DESs

| Variable | First-generation DES (n=375) | | |  | Second-generation DES (n=225) | | |
| --- | --- | --- | --- | --- | --- | --- | --- |
|  | No DM | DM | P value |  | No DM | DM | P value |
|  | n=192 | n=183 |  |  | n=115 | n=110 |  |
| Femoral access | 110(57.3%) | 107(58.5%) | 0.835 |  | 54(47.0%) | 46(41.8%) | 0.503 |
| Multi-vessel PCI | 54(28.1%) | 48(26.2%) | 0.728 |  | 33(28.7%) | 19(17.3%) | 0.057 |
| Mean number of stents | 1.9±0.9 | 2.0±1.0 | 0.294 |  | 1.7±0.8 | 1.7±0.8 | 0.907 |
| PTCA | 14(7.3%) | 9(4.9%) | 0.393 |  | 11(9.6%) | 8(7.3%) | 0.634 |
| PCI failure | 6(3.1%) | 6(3.3%) | 1.000 |  | 7(6.1%) | 5(4.5%) | 0.769 |
| EPD | 4(2.1%) | 2(1.1%) | 0.686 |  | 4(3.5%) | 1(0.9%) | 0.370 |
| Rotational atherectomy | 1(0.5%) | 2(1.1%) | 0.615 |  | 2(1.7%) | 1(0.9%) | 1.000 |
| Aspiration of thrombus | 1(0.5%) | 0(0.0%) | 1.000 |  | 1(0.9%) | 1(0.9%) | 1.000 |
| IVUS | 1(0.5%) | 0(0.0%) | 1.000 |  | 1(0.9%) | 4(3.6%) | 0.205 |
| Medication |  |  |  |  |  |  |  |
| Aspirin | 190(99.0%) | 179(97.8%) | 0.439 |  | 113(98.3%) | 109(99.1%) | 1.000 |
| Statin | 167(87.0%) | 155(84.7%) | 0.556 |  | 101(87.8%) | 97(88.2%) | 1.000 |
| Beta blockers | 149(77.6%) | 143(78.1%) | 1.000 |  | 87(75.7%) | 90(81.8%) | 0.329 |

CABG=coronary artery bypass graft; DES=drug-eluting stent; DM= diabetes mellitus; EPD=embolic protection devices; IVUS= intravascular ultrasound; NCA= native coronary artery; PCI=percutaneous coronary intervention; PTCA= percutaneous coronary angioplasty.

**Table S8** Procedure-related complications of two subgroups of patients with PCI with different generation DESs

| Outcomes | First-generation DES (n=375) | | |  | Second-generation DES (n=225) | | |
| --- | --- | --- | --- | --- | --- | --- | --- |
|  | No DM | DM | P value |  | No DM | DM | P value |
|  | n=192 | n=183 |  |  | n=115 | n=110 |  |
| In-hospital mortality | 0(0.0%) | 1(0.5%) | 0.488 |  | 0(0.0%) | 0(0.0%) | NS |
| Procedural complications |  |  |  |  |  |  |  |
| Dysrhythmia | 0(0.0%) | 2(1.1%) | 0.237 |  | 1(0.9%) | 0(0.0%) | 1.000 |
| Angina in 24h | 6(3.1%) | 9(4.9%) | 0.436 |  | 4(3.5%) | 10(9.1%) | 0.101 |
| Periprocedural MI | 2(1.0%) | 0(0.0%) | 0.499 |  | 1(0.9%) | 5(4.5%) | 0.113 |
| AHF | 1(0.5%) | 1(0.5%) | 1.000 |  | 0(0.0%) | 2(1.8%) | 0.238 |
| Stroke | 0(0.0%) | 1(0.5%) | 0.488 |  | 1(0.9%) | 0(0.0%) | 1.000 |
| Dissection | 1(0.5%) | 2(1.1%) | 0.615 |  | 0(0.0%) | 1(0.9%) | 0.489 |
| Acute closure | 0(0.0%) | 1(0.5%) | 0.488 |  | 0(0.0%) | 1(0.9%) | 0.489 |
| Bleeding | 1(0.5%) | 3(1.6%) | 0.361 |  | 1(0.9%) | 2(1.8%) | 0.615 |

AHF=acute heart failure; CABG=coronary artery bypass graft; DES= drug-eluting stent; DM= diabetes mellitus; NS= not state; MI= myocardial infarction; PCI=percutaneous coronary intervention.

**Table S9** Follow-up outcomes of patients with first-generation DESs PCI

|  | No DM | DM | P value | Unadjusted HR (95% CI) | Adjusted HR (95% CI) |
| --- | --- | --- | --- | --- | --- |
|  | n=192 | n=183 |  |  |  |
| MACEs | 62(32.3%) | 68(37.2%) | 0.193 | 1.26(0.89-1.77) | 1.30(0.88-1.92) |
| Cardiac death | 11(5.7%) | 11(6.0%) | 0.761 | 1.14(.049-2.63) | 1.00(0.39-2.56) |
| MI | 26(13.5%) | 37(20.2%) | 0.055 | 1.63(0.98-2.70) | 1.16(0.66-2.03) |
| HF | 22(11.5%) | 25(13.7%) | 0.334 | 1.33(0.75-2.35) | 1.23(0.63-2.41) |
| Revascularization | 34(17.7%) | 34(18.6%) | 0.700 | 1.10(0.68-1.77) | 1.27(0.74-2.17) |

CABG=coronary artery bypass graft; DES= drug-eluting stent; DM= diabetes mellitus; HF=acute heart failure; MACEs= major adverse cardiac events; MI= myocardial infarction.; HR=hazard ratio; CI= conference interval.

**Table S10** Follow-up outcomes of patients with second-generation DESs PCI

|  | No DM | DM | P value | Unadjusted HR (95% CI) | Adjusted HR (95% CI) |
| --- | --- | --- | --- | --- | --- |
|  | n=115 | n=110 |  |  |  |
| MACEs | 28(24.3%) | 44(40.0%) | 0.016 | 1.78(1.11-2.86) | 1.76(1.00-3.08) |
| Cardiac death | 5(4.3%) | 5(4.5%) | 0.852 | 1.13(0.33-3.89) | 1.10(0.01-1.47) |
| MI | 9(7.8%) | 18(16.4%) | 0.038 | 2.28(1.02-5.07) | 1.90(0.75-4.81) |
| HF | 8(7.0%) | 19(17.3%) | 0.018 | 2.62(1.15-5.99) | 1.87(0.72-4.89) |
| Revascularization | 17(14.8%) | 22(20.0%) | 0.305 | 1.39(0.74-2.62) | 1.67(0.79-3.53) |

CABG=coronary artery bypass graft; DES= drug-eluting stent; DM= diabetes mellitus; HF=acute heart failure; MACEs= major adverse cardiac events; MI= myocardial infarction; HR=hazard ratio; CI= conference interval.

**Table S11** Follow-up outcomes of patients with incomplete revascularization vs complete revascularization

| Outcomes | IR | CR | P value |
| --- | --- | --- | --- |
|  | n=37 | n=687 |  |
| MACEs | 15(40.5%) | 227(33.0%) | 0.373 |
| Cardiac death | 3(8.1%) | 33(4.8%) | 0.421 |
| MI | 8(21.6%) | 97(14.1%) | 0.228 |
| HF | 1(2.7%) | 86(12.5%) | 0.299 |
| Revascularization | 5(13.5%) | 127(18.5%) | 0.661 |

CR= complete revascularization; HF=acute heart failure; IR=incomplete revascularization; MACEs= major adverse cardiac events; MI= myocardial infarction.
