# Supplementary figures and images for: Percutaneous coronary intervention in diabetic versus non-diabetic patients with prior coronary artery bypass grafting: a propensity score matching study
Source: BMC Cardiovasc Disord. 2020 Apr 6;20:159. doi: 10.1186/s12872-020-01447-8 (PMC7137249; doi:10.1186/s12872-020-01447-8)

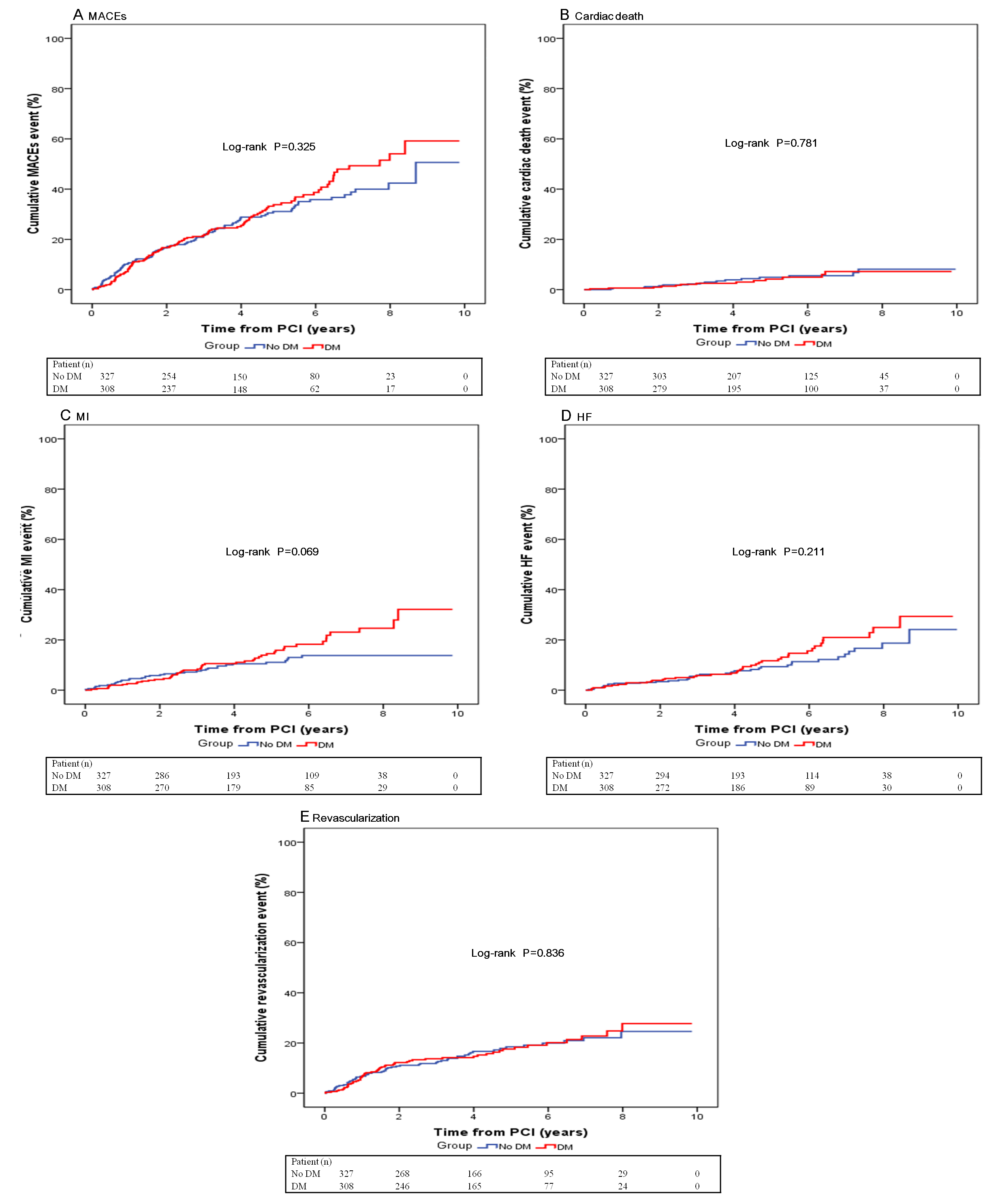

Supplement: Supplementary file 2 — Additional file 2: Figure S1. Incidence of MACEs (A), cardiac death (B), MI(C), HF(D) or revascularization (E) of patients with PCI in only NCA (No DM vs DM) using the Kaplan-Meier method. P value was calculated by log-rank test. DM = diabetes mellitus, HF = acute heart failure, NCA = native coronary artery, MACEs = major adverse cardiac events, MI = myocardial infarction. [file 12872_2020_1447_MOESM2_ESM.tif]

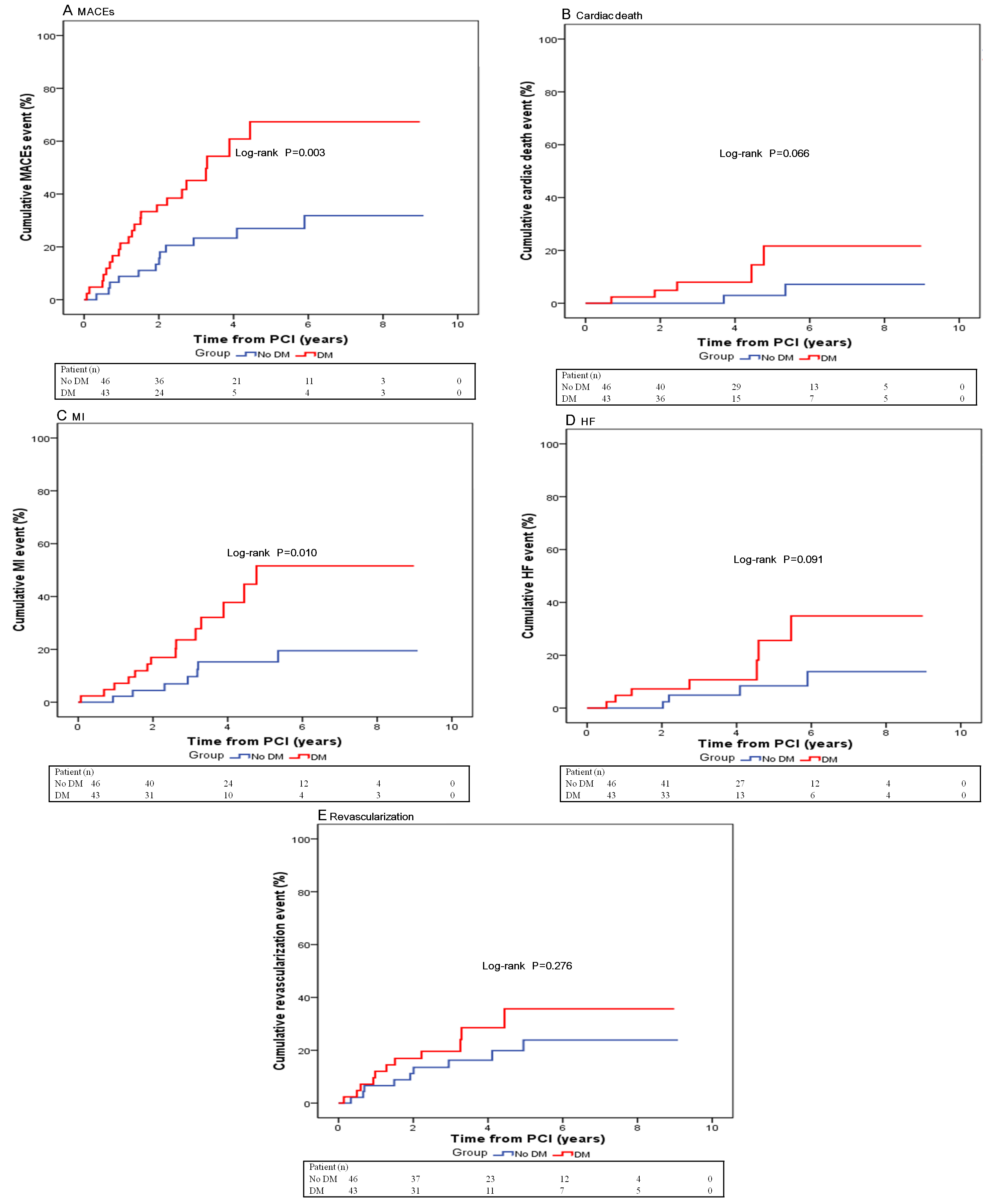

Supplement: Supplementary file 3 — Additional file 3: Figure S2. Incidence of MACEs (A), cardiac death (B), MI(C), HF(D) or revascularization (E) of patients with PCI in NCA and graft (No DM vs DM) using the Kaplan-Meier method. P value was calculated by log-rank test. DM = diabetes mellitus, HF = acute heart failure, NCA = native coronary artery, MACEs = major adverse cardiac events, MI = myocardial infarction. [file 12872_2020_1447_MOESM3_ESM.tif]

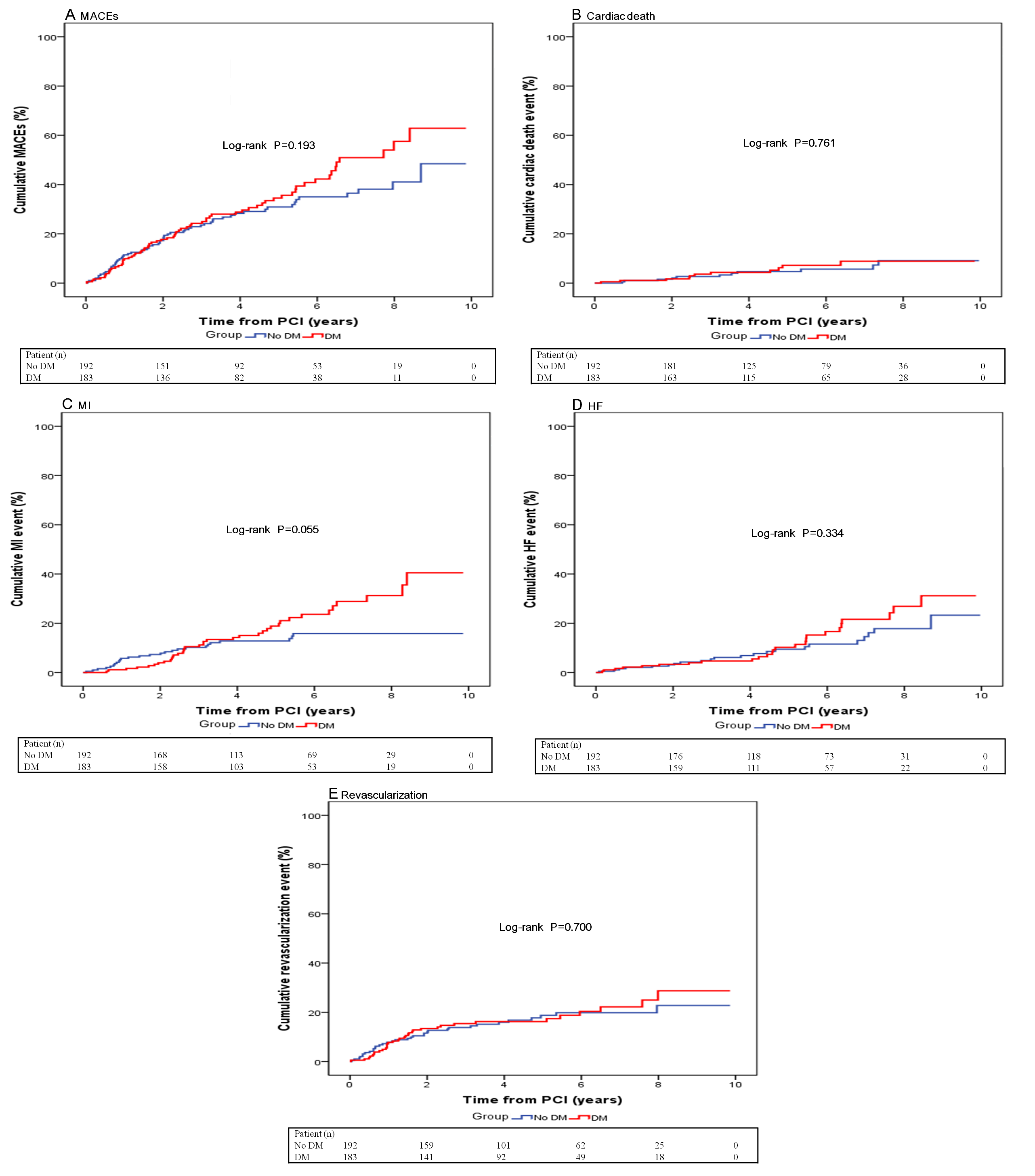

Supplement: Supplementary file 4 — Additional file 4: Figure S3. Incidence of MACEs (A), cardiac death (B), MI(C), HF(D) or revascularization (E) of patients with first-generation DES PCI (No DM vs DM) using the Kaplan-Meier method. P value was calculated by log-rank test. DM = diabetes mellitus, HF = acute heart failure, NCA = native coronary artery, MACEs = major adverse cardiac events, MI = myocardial infarction. [file 12872_2020_1447_MOESM4_ESM.tif]

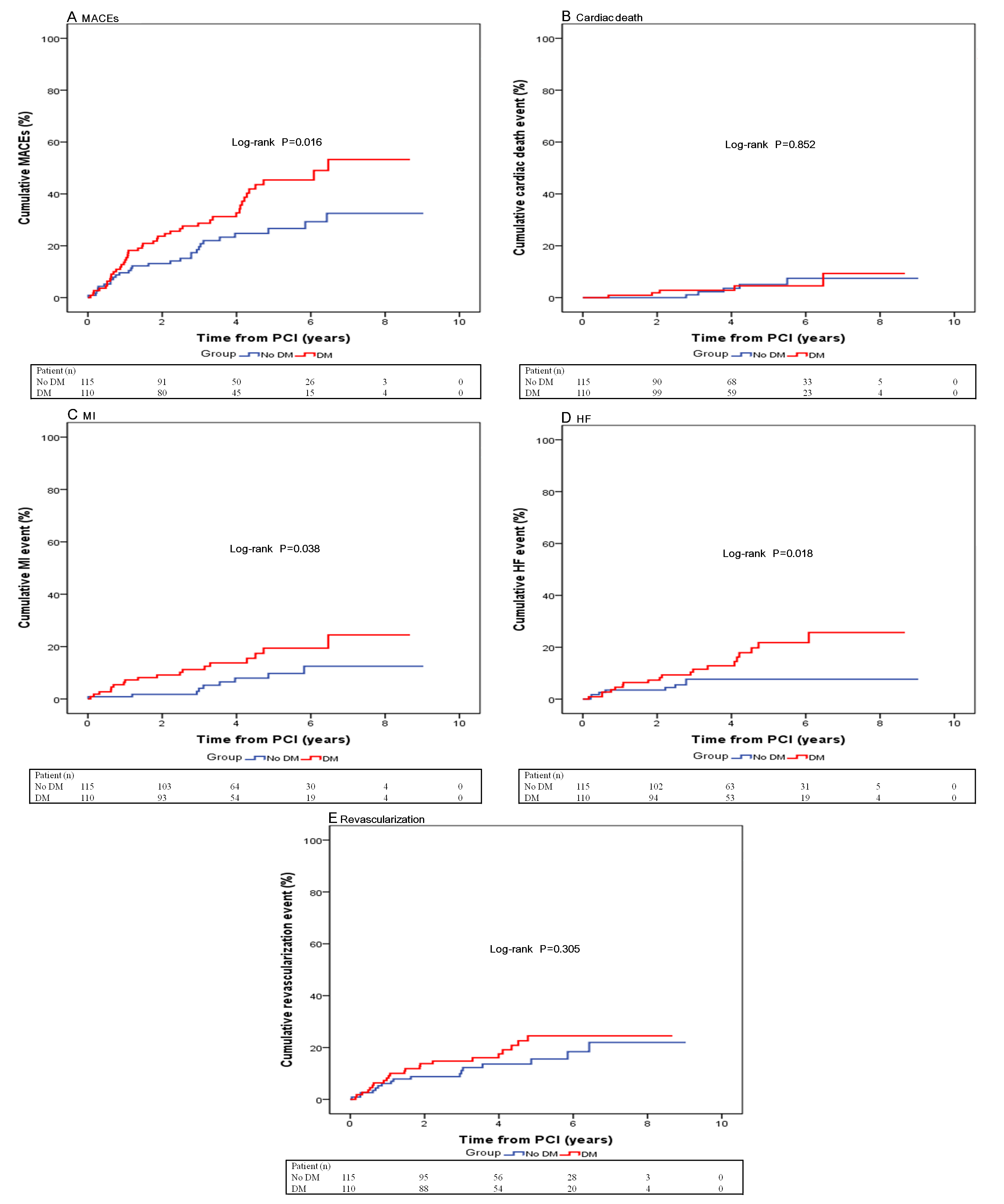

Supplement: Supplementary file 5 — Additional file 5: Figure S4. Incidence of MACEs (A), cardiac death (B), MI(C), HF(D) or revascularization (E) of patients with second-generation DES PCI (No DM vs DM) using the Kaplan-Meier method. P value was calculated by log-rank test. DM = diabetes mellitus, HF = acute heart failure, NCA = native coronary artery, MACEs = major adverse cardiac events, MI = myocardial infarction. [file 12872_2020_1447_MOESM5_ESM.tif]
